# Gross Motor Skills Predict Classroom Behavior in Lower-Income Children

**DOI:** 10.3389/fspor.2019.00029

**Published:** 2019-09-18

**Authors:** Ryan D. Burns, Wonwoo Byun, Timothy A. Brusseau

**Affiliations:** Physical Activity Research Lab, Department of Health, Kinesiology, and Recreation, University of Utah, Salt Lake City, UT, United States

**Keywords:** behavior, child, gross motor skills, physical activity, schools

## Abstract

Children from lower income families tend to have low levels of on-task behavior in the academic classroom. The purpose of this study was to examine the associations of gross motor skills and classroom behavior in a sample of lower-income children. Participants were a sample of 1,135 school-aged children (mean age = 8.3 ± 1.8 years) recruited from three low-income US schools. A reduced version of the Test for Gross Motor Development 2nd Edition (TGMD-2) was used to assess gross motor skills. Total TGMD-2 scores, locomotor subtest scores, and object control subtest scores were stratified into quintiles for analysis. Students' classroom behavior was recorded 1 year later using a Planned Activity Check (PLACHECK) 5-s momentary time sampling procedure. Classrooms were dichotomized into those that had students at least 80% on-task and those that did not. Multilevel generalized mixed models were employed to examine the relationship between gross motor skills and meeting at least 80% classroom behavior, adjusting for age, sex, and change in BMI, and aerobic fitness. Children in the highest TGMD-2 quintile had 4.17 higher odds of being in an on-task classroom 1 year later (95%CI [2.25–7.76], *p* < 0.001). This relationship was primarily driven by the relationship between object control quintile scores and classroom behavior, as children within the higher quintile for object control had 3.81 higher odds of being in an on-task classroom 1 year later (95%CI [2.67–5.46], *p* < 0.001). There was a significant relationship between individual gross motor skills, specifically object control skills, and group level on-task classroom behavior in lower-income children.

## Introduction

Elementary school children who sit through prolonged academic instruction time can become restless and experience reduced concentration, leading to disruptive classroom behavior (Pellegrini and Davis, 1993). Furthermore, off-task classroom behaviors may lead to reduced memory retention, leading to poorer academic performance (Blankenship et al., 2015). Persistent disruptive behavior may also negatively impact classmates' classroom behavior and academic performance and place unnecessary burdens on academic teachers (Burke et al., 2011). It has been recommended that a classroom should not be off-task for more than 20% of class time to facilitate an optimal learning environment (Rathvon, 2008). Therefore, behavioral strategies to achieve this threshold are important to maintain a classroom environment that is conducive to learning.

School-based programming to improve physical activity has been shown to improve classroom behavior (Mahar et al., 2006; Stylianou et al., 2016). Acute and habitual physical activity may provide a means to moderate psychological arousal by lowering anxiety (Anderson and Shivakumar, 2013) and habitual physical activity may moderate physiological arousal by decreasing stressor-induced cortisol reactivity (Puterman et al., 2011), which may have a positive effect on classroom behavior in children. Studies have shown that physical activity interventions lead to improved classroom behavior in both healthy elementary school-aged children and in children with behavioral health disorders (Bowling et al., 2017; Harvey et al., 2018). Thus, identifying different ways to improve physical activity may be an effective strategy to facilitate better learning environments. Habitual and acute physical activity and exercise may improve attention span and working memory by altering the neurochemicals serotonin, dopamine, norepinephrine, in addition to brain-derived neurotrophic factor, synaptic proteins, and insulin-like growth factor-1 (Winter et al., 2007; Hsieh et al., 2018). Hyperactivity in students and its associated disruptive behaviors in classroom settings may also be attenuated by both habitual and acute physical activity's ability to improve inhibitory control (Berwid and Halperin, 2012). The acute effect of physical activity on neurocognitive function seems to be dose-dependent, in that physical activity and exercise of higher intensities have a more significant beneficial impact on tests of cognitive function (Gonweld et al., 2018). Improving both habitual and acute higher intensity physical activity behaviors and its subsequent links to improved classroom behavior may be facilitated by improving correlates of physical activity.

A correlate of physical activity is gross motor skills (Loprinzi et al., 2015; Burns and Fu, 2018). Fundamental gross motor skills grow from rudimentary phases of infancy to more complicated movements that serve as building blocks for complex movements (Burton and Miller, 1998). In addition to individual age and maturation (Moller et al., 2008), behavioral factors such as physical activity play a role in gross motor skill development. Specifically, higher levels of gross motor skills associate with higher levels of moderate-to-vigorous physical activity (Silva-Santos et al., 2019). Mechanisms for these links include gross motor skills helping children control their bodies, manipulate their environment, and form complex skills involved in sports and other recreational activities (Davis and Burton, 1991). Physical activity interventions have shown to have a positive effect on gross motor skills in children (Robinson et al., 2016). However, conceptual models have proposed and empirical research has shown that gross motor competency also improves habitual physical activity in children, providing evidence for a potential bidirectional relationship between the two constructs (Robinson et al., 2015).

Stodden et al. (2008) proposed a conceptual framework linking improvements in gross motor skills with improvements in physical activity in children and adolescents, which will further lead to decreases in obesity and cardio-metabolic disease risk. Specifically, higher levels of gross motor competency would promote higher levels of physical activity throughout childhood. These relationships lead to a positive spiral of engagement where achievement of a healthy weight would lead to sustained motor competence and maintenance of higher levels of physical activity. Many of these relationships have been empirically tested and may be partially mediated through specific domains of health-related fitness in addition to salient psychosocial variables (Barnett et al., 2008, 2011; Burns et al., 2017). Because motor competence is a primary antecedent in this conceptual model, and because of the established relationships between physical activity with classroom behavior, it is theoretically plausible that higher levels of gross motor skills will also correlate with improved classroom behavior in children. Theoretical mediators of this relationship may be higher habitual physical activity. However, to the authors' knowledge, the correlation between gross motor skills and classroom behavior has not been thoroughly investigated.

A population that can benefit from improved classroom behaviors is children from lower income families. Low family income is associated with poor academic achievement among children (Reardon, 2011). Factors mediating the relationship between low-income status and poor academic underachievement include a lack of support and a lack of a positive learning environment (Banerjee, 2016). Positive learning environments associate with sufficient amounts of on-task classroom behavior (Scott et al., 2007). Furthermore, low income children tend to have lower levels of physical activity (Gordon-Larsen et al., 2006; Jin and Jones-Smith, 2015). As a result, physical activity interventions have been derived with a primary aim to facilitate better academic performance (Centers for Disease Control and Prevention, 2013; Hillman et al., 2014). Because gross motor skills may lead to higher levels of physical activity, and physical activity may lead to better classroom behavior via psychological and physiological arousal modulation, a link between gross motor skills and better classroom behavior is possible but has been largely unexamined within the current literature. Therefore, the purpose of this study was to examine the associations of individual gross motor skills with the probability of a child belonging to a classroom that was sufficiently on-task 1 year later. It was hypothesized that higher levels of individual gross motor skills will associate with a child belonging to a sufficiently on-task homeroom academic classroom 1 year later.

## Methods

### Participants

The final sample consisted of participants were a convenience sample of 1,135 school-aged children, which consisted of children with complete data. Children were recruited from the K-5th grades from three low-income schools located in low socio-economic status neighborhoods from the Mountain West Region of the US. A sensitivity analysis for logistic regression was conducted using G*Power which showed that with our sample size (*N* = 1,135), a two-tailed alpha level of 0.05, and power at 80%, the minimum detectable effect is an Odds Ratio = 1.23. All three schools were recruited from the same urban school district. The mean age for the sample at baseline (year 1) was 8.3 ± 1.8 years (Age range: 6–12 years) and there were 569 girls and 566 boys who participated. Age was self-reported from the participants and no exact birthdates were collected. Given the information presented on the school district website, over 78% of the student enrollment at each of the three schools belonged to an ethnic minority, and over 91% of the students were characterized as low-income, receiving free or reduced lunch prices. Low income families are defined by household income that is below twice the federal poverty threshold. In 2015, the federal poverty threshold for a family of four with two children was $24,036. Individual-level race/ethnicity and socio-economic status data was not collected directly from the children. Participant exclusion criteria was any condition precluding child participation in gross motor skill testing and fitness testing. The child also had to be enrolled in one of the three recruited schools for at least 2 years. No special needs children were recruited and special needs classes were not observed for classroom behavior. Written assent and consent were obtained from all observed students and written consent was obtained from the parents prior to data collection. School district approval was also obtained. The University Institutional Review Board approved the protocols employed in this study.

### Classroom Behavior Assessment

Classroom behavior data was recorded directly in the classroom setting by both a primary and secondary observer. Time of day, content, and teaching methodology varied across classrooms, and were not controlled for due to classroom observation access scheduling. Students' on-task and off-task behavior was recorded using Planned Activity Check (PLACHECK) 5-s momentary time sampling. Behavioral observations occurred at the end of a 5-s time interval, which commenced after the observer marked the behavior from the immediately prior interval (van der Mars, 1989). Only behavior at the end of a 5-s interval were recorded. Each interval was coded as being on-task or off-task. The primary and secondary observers established the order of observation sequence prior to starting observations. Observations were made from a left to right sequence in the classroom during each observation period, lasting 15 min. Observations commenced 5 min from the start of the lesson. Observers observed a respective student for 5 s before moving on to the next student in the sequence. Students within the sequence were observed multiple times per observation session. A prerecorded audio file signaled the start of each 5-s interval to the observers. Upon hearing the 5-s signals, observers observed and recorded classroom behavior. A primary observer recorded all observations in this study and a secondary observer recorded approximately 50% of the classes with the primary observer to determine interobserver reliability. Inter-observer reliability was calculated by taking the two observers' agreements of on-task behavior and off-task behavior divided by the total number if observations. To obtain a percentage, the agreement proportion was multiplied by 100. The inter-observer reliability was found to be 90%. The aforementioned procedures are in accordance to those recommended by Mahar (2011). Training for the procedures involved having the observers watch a video of a recorded 3rd grade classroom lesson to practice observing and recording behaviors in approximately 15-min intervals. The training was conducted 1–2 weeks prior to the start of data collection. It has been suggested that a classroom should display at or above 80% on-task behavior to sustain an academic environment conducive for optimal learning (Michem et al., 2001). Therefore, classrooms were stratified into those that had at least 80% of the students on-task during an observation period and those that did not.

### Gross Motor Skill Assessment

A reduced version of the Test for Gross Motor Development 2nd Edition (TGMD-2) was used to assess gross motor skills (Ulrich, 2000). Because of the time constraints, not all TGMD-2 testing items were assessed during a single 50-min physical education class. One-half (6/12) of the TGMD-2 items were administered. The locomotor subtest items included running, skipping, and leaping. The object control subtest items included throwing, catching, and kicking. Items were chosen based on available gym space and equipment in addition to relevance to common activities engaged in during physical education. Each student performed the test items across two trials that were each scored based on specific performance criteria (0 = did not perform correctly; 1 = performed correctly; Ulrich, 2000). The locomotor and ball subtest scores were both out of 11 and the total TGMD-2 scores were out of 22. The content sampling, time sampling, and inter-scorer differences of the TGMD-2 was determined to be acceptable with coefficients of 0.87, 0.88, and 0.98, respectively (Ulrich, 2000). The TGMD-2 demonstrated good content-description, criterion-prediction, and construct-identification validity evidence using a sample of 1,208 persons in 10 states from the US (Ulrich, 2000).

### Health-Related Fitness Assessment

Body Mass Index (BMI) was calculated using standard procedures taking a student's weight in kilograms divided by the square of height in meters (Nuttall, 2011). Height was measured to the nearest 0.01 meters using a portable stadiometer (Seca 213; Hanover, MD, USA) and weight was measured to the nearest 0.1 kilogram using a portable medical scale (BD-590; Tokyo, Japan). Height and weight were collected in a private room during each student's physical education class.

Cardiorespiratory endurance was assessed using the 20-m Progressive Aerobic Cardiovascular Endurance Run (PACER). PACER was administered during each student's physical education class. PACER was conducted on a marked gymnasium floor with background music provided by a compact disk. Each student was instructed to run from one floor marker to marker across a 20-m distance within an allotted time frame. PACER was performed in two sex-specific groups. The allotted time given to reach the specified distance incrementally shortened as the test progressed. If the student twice failed to reach the other floor marker, the test was terminated and the final score was recorded in laps. PACER has shown moderate criterion-related validity scores for predicting aerobic capacity in children (Mayorga-Vega et al., 2015).

### Procedures

Gross motor skills were collected during year 1 (Spring, 2015) and BMI and PACER were collected across successive weeks during year 1 and year 2 (Spring, 2015, 2016). A total of 63 classrooms were observed for classroom behavior in year 2 (Spring, 2016). Students from year 1 were matched to a respective classroom during year 2. Homerooms were used for the collection of all classroom behavior data.

### Data Analysis

Data were screened for outliers using boxplots and *z*-scores and checked for Gaussian distributions using k-density plots. To describe the sample, differences between sexes on all observed variables were examined using independent *t*-tests. Sex difference pairwise comparison effect sizes were calculated using Cohen's delta (*d*) where *d* < 0.20 indicating a small effect size, *d* = 0.50 indicating a medium effect size, and *d* > 0.80 indicating a large effect size (Cohen, 1988). Predictive utility of TGMD-2 quintile scores on students belonging to classroom that was at least 80% on-task was examined using multilevel generalized mixed effects models. Quintiles were used instead of continuous scores to facilitate interpretation of the odds ratios from logistic regression using the lowest quintile as the reference level. Random intercepts were employed at the school level to adjust for clustering within the data structure. The school random intercept was the only random effect. Fixed effects were the gross motor skill quintile categories and the age, sex, and change in BMI and PACER covariates (potential confounders). Predictors were entered into the model using block-wise entry. The first block (Model 1) consisted of the TGMD-2 quintile scores. The second block (Model 2) consisted of Model 1 plus the potential confounders of age, sex, change in BMI (year 1–year 2), and change in PACER laps (aerobic fitness; year 1–year 2). Reporting of the results consisted of communicating the odds ratios (*ORs*) with 95% Confidence Intervals (95% CIs). For predictors yielding significant effects, marginal probability plots were derived to graphically display the relationships. Secondary analyses were conducted to determine whether the locomotor and object control subtest quintile subtest scores were associated with a student belonging to a sufficiently on-task classroom. All analyses had an initial alpha level of *p* < 0.05 and were carried out using STATA v15.0 statistical software package (StataCorp, College Station, Texas, USA).

## Results

**Figure 1:**
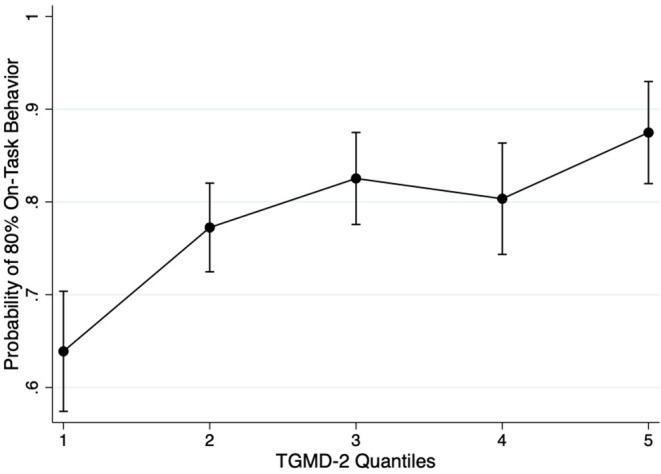
Marginal probability plot showing the relationship between TGMD-2 Quantiles and the probability of a classroom achieving at least 80% on-task classroom behavior. TGMD-2 stands for the Test for Gross Motor Development 2nd Edition; error bars are 95% Confidence Intervals.

Descriptive statistics are reported in Table 1. Boys had higher PACER laps at year 1 and 2, TGMD-2 total scores, locomotor subtest scores, and object control scores during year 1 compared to girls. Locomotor skill sex differences were driven by the running test; object control sex differences were driven by all three object control testing items. Sex differences were generally characterized by small effect sizes. Twenty students with complete data at year 1 were lost to follow up at year 2 due to transferring to another school; therefore, classroom behavior data was not collected. Parameter estimates from the generalized mixed effect model are reported in Table 2. TGMD-2 quintiles significantly predicted whether or not a student belonged to a sufficiently on-task behavior classroom across all categories (Model 1; *p* < 0.05). This observed significant relationship was held even after adjusting for the potential confounders of age, sex, and change in BMI and aerobic fitness (Model 2; *p* < 0.05). The strongest observed relationship was between children within the highest TGMD-2 quintile having 4.17 higher odds of being in an on-task classroom 1 year later (95%CI [2.25–7.76], *p* < 0.001). This relationship was driven by the relationship between object control subtest quintile scores and classroom behavior, as children within the higher quintile for object control had 3.81 higher odds of being in an on-task classroom 1 year later (95%CI [2.67–5.46], *p* < 0.001). Secondary analyses showed that locomotor subtest quintile scores did not significantly predict a student belonging to an on-task classroom. Figure 1 displays the marginal predicted probabilities of students belonging to an on-task behavior classroom as a function of TGMD-2 quintile scores. All multilevel models adjusted for the clustering of students within schools.

**Table 1:** Descriptive statistics (means and standards deviations).

	**Total sample (*N* = 1,135)**	**Boys (*n* = 566)**	**Girls (*n* = 569)**	**Sex difference (95% Confidence Interval)**	**Sex difference Cohen's *d***
Baseline Age (years)	8.3 (1.8)	8.3 (1.8)	8.3 (1.8)	0.0 (−0.2–0.2)	0.0
Year 1 Height (m)	1.33 (0.14)	1.33 (0.14)	1.33 (0.14)	0.0 (−0.02–0.02)	0.0
Year 2 Height (m)	1.36 (0.11)	1.35 (0.14)	1.36 (0.14)	0.0 (−0.02–0.02)	0.01
Year 1 Weight (kg)	33.7 (13.2)	33.7 (13.1)	33.6 (13.3)	0.2 (−1.3–1.7)	0.01
Year 2 Weight (kg)	33.8 (13.4)	33.7 (13.2)	33.9 (13.6)	1.2 (−2.4–4.7)	0.03
Year 1 BMI (kg/m^2^)	18.6 (6.0)	18.6 (6.0)	18.5 (5.9)	0.1 (−0.6–0.8)	0.02
Year 2 BMI (kg/m^2^)	17.7 (4.7)	17.6 (4.3)	17.8 (5.1)	0.2 (−0.9–1.1)	0.01
Year 1 PACER laps	24.6 (13.7)	**25.6^†^** **(14.7)**	23.6 (12.6)	2.0 (0.4–3.6)	0.15
Year 2 PACER laps	29.8 (18.0)	**31.3^†^** **(19.6)**	28.3 (16.1)	3.0 (1.0–5.1)	0.16
Running (out of 4)	3.5 (0.6)	**3.7 (0.6)**	3.5 (0.6)	0.2 (0.1–0.3)	0.17
Skipping (out of 4)	2.5 (0.6)	2.5 (0.6)	2.5 (0.6)	0.0 (−0.1–0.06)	0.0
Leaping (out of 3)	2.2 (0.6)	2.2 (0.6)	2.2 (0.6)	0.0 (−0.1–0.06)	0.0
Catching (out of 3)	2.6 (0.8)	**2.8^†^** **(0.7)**	2.5 (0.8)	0.3 (0.2–0.4)	0.41
Kicking (out of 4)	3.0 (0.9)	**3.1^†^** **(0.8)**	2.8 (0.8)	0.3 (0.2–0.4)	0.39
Overhead throwing (out of 4)	2.9 (0.8)	**3.0^†^** **(0.8)**	2.8 (0.8)	0.2 (0.1–0.3)	0.27
TGMD-2 scores (out of 22)	16.7 (3.8)	**17.2^†^** **(3.7)**	16.1 (3.9)	1.1 (0.7–1.5)	0.29
Locomotor skills (out of 11)	8.1 (2.0)	**8.2^†^** **(2.0)**	8.0 (2.1)	0.2 (0.02–0.5)	0.11
Object control skills (out of 11)	8.4 (2.2)	**8.8^†^** **(2.0)**	8.0 (2.3)	0.8 (0.6–1.1)	0.38

BMI stands for Body Mass Index; PACER stands for Progressive Aerobic Cardiovascular Endurance Run; TGMD-2 stands for Test for Gross Motor Development-2nd Edition; bold and

† *denotes statistical significance, p < 0.05*.

**Table 2:** Fixed-effect parameter estimates and random effects from the multilevel generalized mixed effects model.

		**Model 1**		**Model 2**	
		***OR***	**95% CI**	***OR***	**95% CI**
Fixed effects	TGMD-2 1st Quintile	Reference		Reference	-
	TGMD-2 2nd Quintile	**2.32^†^**	1.59–3.38	**1.98^†^**	1.34–2.93
	TGMD-2 3rd Quintile	**3.57^†^**	2.32–5.50	**2.76^†^**	1.74–4.39
	TGMD-2 4th Quintile	**3.97^†^**	2.53–6.20	**2.40^†^**	1.42–4.04
	TGMD-2 5th Quintile	**7.62^†^**	4.41–13.17	**4.17^†^**	2.25–7.76
	Baseline Age (years)			**1.32^†^**	1.18–1.48
	Sex (boy referent)			**1.44^†^**	1.06–1.94
	Change in PACER laps			0.99	0.98–1.01
	Change in BMI (kg/m^2^)			0.99	0.95–1.05
School random effect	Intercept	0.00	0.00–0.00	0.00	0.00–0.00

Model 1 includes the TGMD-2 predictor; Model 2 is the Model 1 and the potential confounding of age, sex, and change in PACER and BMI; OR stands for Odds Ratio; 95% CI stands for 95% Confidence Interval; BMI stands for Body Mass Index; PACER stands for Progressive Aerobic Cardiovascular Endurance Run; TGMD-2 stands for Test for Gross Motor Development-2nd Edition; bold and

† *indicates statistical significance, p < 0.05*.

## Discussion

The purpose of this study was to examine the relationship between individual gross motor skills and classroom behavior observed 1-year later in a sample of children from lower-income schools. The results indicated that there is a relationship between individual gross motor skills associating with whether or not a child belonged to a sufficiently on-task classroom 1 year later. Secondary analyses indicated that this relationship was primarily driven by object control scores. Results from this prospective analysis provides information for researchers and practitioners with the aim to improve classroom behavior in lower income children. The results also may direct avenues for additional research examining these relationships via moderated and mediated mechanisms.

The salient finding from the current study was that individual gross motor skills, specifically object control skills, significantly associated with whether or not child belonged to a classroom that was sufficiently on-task 1 year later. Although health-related fitness has been shown to associate with classroom behavior in the pediatric population (Davis and Cooper, 2011; Finn et al., 2018), given the lack of significance in the relationship between BMI and PACER score change and classroom behavior, the relationship between gross motor skills and classroom behavior theoretically may have been mediated by physical activity. What may be important in the observed relationships the type of physical activity engaged in, as previous research has shown that participation in externally paced sports associates with better performance on tests of vigilance capacity, independent of an athletes' level of aerobic fitness (Ballester et al., 2019; Sanabria et al., 2019). The relationship between gross motor skills and physical activity has also previously been established (Barnett et al., 2016). Stodden et al. (2008) developed a conceptual model that clearly links these constructs and specifies the moderating effects of developmental age in the direction of inferred causation. Several studies conducted by Barnett and colleagues support these longitudinal relationships, especially using object control/ball skills subtest scores, possibly because of the high prevalence of locomotor skill proficiency among older children (Barnett et al., 2008, 2011, 2016). Although most studies focus on the links among gross motor skills, physical activity, and health outcomes, a recent systematic review has shown that physical activity effects both gross motor skills and cognitive development in preschool-aged children (Zeng et al., 2017). Because of the multifactorial benefits of physical activity on health-related outcomes (Ahn et al., 2018; Tarp et al., 2018), and because physical activity has also shown a beneficial effect on cognitive functioning in children (Castelli et al., 2011; Donnelly and Lambourne, 2011; Donnelly et al., 2016; Bidzan-Bluma and Lipowska, 2018), extending these conceptual models linking gross motor skills to physical activity to variables within the affective and cognitive domains is defensible. Despite this theoretical plausibility, studies examining the relationships between gross motor skills and affective and cognitive outcomes is sparse. It has been found that fine motor skills and executive function can predict kindergartener's academic achievement (Cameron et al., 2012). In younger children aged 3–5 years old, children with better object control manipulation skills in the fall showed significantly stronger social behavior in the classroom in the spring (MacDonald et al., 2016). However, studies showing the prospective link between gross motor skills and classroom behavior in older children are lacking.

Because of the results of the current study and the potential bidirectionality of the relationship between gross motor skills and physical activity, targeting gross motor skill development during health-based programming may potentially elicit improvements in classroom behavior (Lopes et al., 2013). Motor incompetent children suffer from emotional and cognitive issues that reduce quality of life (Cairney et al., 2010). Disengagement in physical activity participation because of the lack of gross motor skills development may also affect health outcomes as motor incompetent children track into adolescence and into adulthood. Unfortunately, lower income children tend to have motor incompetency and may slowly develop specific fundamental skills needed for physical activity engagement (Goodway et al., 2010; Morley et al., 2015; Playford et al., 2017). The results of this study show that improving gross motor skills may lead to improved classroom behavior in lower income children, which may further lead to improved academic outcomes and overall quality of life.

The results from this study yield avenues for future research. Future research could use accelerometer-assessed physical activity as a mediator variable within the relationship between gross motor skills and classroom behavior. Using accelerometer data, the constructs of sedentary times and physical activity can be analyzed within a time-use approach using compositional data analysis (Burns et al., 2019). This could provide a clearer understanding on the relationships among sedentary times, physical activity, gross motor skills and classroom behavior by dividing time-use behaviors into distinct compositional parts. The relationship between gross motor skills classroom behavior may also be mediated through psychosocial constructs that align with Social-Cognitive Theory and Self-Determination Theory (Barnett et al., 2008; De Meester et al., 2016; Fu and Burns, 2018). The constructs of enjoyment, perceived competence, and self-efficacy may be within the potential causal pathway between gross motor skills and academic classroom behavior. Testing both time-use behaviors (i.e., sedentary times and physical activity) and psychosocial constructs together may provide a more comprehensive approach to understand mediated pathways between gross motor skills and on-task classroom behavior (Stodden et al., 2014). There may also be moderators within these relationships such as socioeconomic status and especially maturation stage that should be explored with additional research as developmental maturation links with both gross motor development, especially ball skills, and classroom behavior. Controlling for factors related to the classroom lesson (e.g., topic, time of day, teaching method, etc.) should also be explored. Finally, collecting motor skill and classroom behavior data on multiple repeated measures time-points across multiple years may provide greater evidence for causation.

There are limitations that should be considered from this study. First, our findings may have limited generalizability because the sample consisted of lower income children recruited from 3 schools from the Mountain West region of the US. Second, the unit of measurement for classroom behavior was analyzed at the classroom level but gross motor skills were assessed at the student level; therefore, caution must be exercised for interpreting findings from this study at the individual level. Third, the data collection was prospective with two time-points, causal inferences may be stronger if a greater number of repeated measures time-points were observed. Fourth, student behavior could have been modified due to the presence of observers within the classroom. Fifth, only chronological age was used as a covariate to control for potential confounding. The use of maturation stage would have been more relevant. Additionally, BMI was also used as a covariate. Use of percent body fat may have been more relevant in younger children (Taylor et al., 1997). Finally, aforementioned moderators and mediators of effect were not tested and should be a priority in future research.

In conclusion, individual gross motor skills, specifically object control skills, related to group level classroom behavior assessed 1 year later in a sample of children from lower income schools. This relationship was independent of child age, sex, and change in BMI and aerobic fitness. Given Stodden's conceptual framework, a possible mechanism for these relationships is the established relationship between gross motor skills and physical activity, which itself is known to correlate with classroom behavior. This study provides some evidence that higher levels of gross motor skills could relate to improved odds of on-task classroom behavior in a sample of low-income children. However, because motor skill data were collected on the individual level and classroom behavior data were collected on the class level, this relationship remains largely speculative. The results potentially provide evidence of the benefits of gross motor skills within the affective and cognitive domains in children.

## Data Availability

The datasets generated for this study are available in the Supplementary Material.

## Ethics Statement

This studies involving human participants were reviewed and approved by University of Utah Institutional Review Board. Written informed consent to participate in this study was provided by the participants' legal guardian/next of kin.

## Author Contributions

RB conceived the study, performed the data analysis, wrote the initial draft of the manuscript, and approved the final version of the manuscript to be submitted. WB and TB conceived the study, wrote the initial draft of the manuscript, and approved the final version of the manuscript to be submitted.

### Conflict of Interest Statement

The authors declare that the research was conducted in the absence of any commercial or financial relationships that could be construed as a potential conflict of interest.

## References

[B1] Ahn J. V. Sera F. Cummins S. Flouri E. (2018). Associations between objectively measured physical activity and later mental health outcomes in children: findings from the UK millennium cohort study. J. Epidemiol. Commu. Health. 72, 94–100. 10.1136/jech-2017-209455 29183954

[B2] Anderson E. Shivakumar G. (2013). Effects of exercise and physical activity on anxiety. Front. Psychiatry 4:27. 10.3389/fpsyt.2013.00027 23630504 PMC3632802

[B3] Ballester R. Huertas F. Pablos-Abella C. Llorens F. Pesce C. (2019). Chronic participation in externally paced, but not self-paced sports is associated with the modulation of domain-general cognition. Eur. J. Sport Sci. 19, 1110–1119. 10.1080/17461391.2019.1580318 30786834

[B4] Banerjee P. A. (2016). A systematic review of factors linked to poor academic performance of disadvantaged students in science and maths in schools. Cogent Edu. 3:1178441 10.1080/2331186X.2016.1178441

[B5] Barnett L. M. Lai S. K. Veldman S. L. C. Hardy L. L. Cliff D. P. Morgan P. J.. (2016). Correlates of gross motor competence in children and adolescents: a systematic review and meta-analysis. Sports Med. 46, 1663–1688. 10.1007/s40279-016-0495-z 26894274 PMC5055571

[B6] Barnett L. M. Morgan P. J. Van Beurden E. Ball K. Lubans D. R. (2011). A reverse pathway? Actual and perceived skill proficiency and physical activity. Med. Sci. Sports Exerc. 43, 898–904. 10.1249/MSS.0b013e3181fdfadd 20962694

[B7] Barnett L. M. Morgan P. J. Van Beurden E. Beard J. R. (2008). Perceived sports competence mediates the relationship between childhood motor skill proficiency and adolescent physical activity and fitness: a longitudinal assessment. Int. J. Behav. Nutrit. Phys. Act 5:40. 10.1186/1479-5868-5-40 18687148 PMC2569960

[B8] Berwid O. G. Halperin J. M. (2012). Emerging support for a role of exercise in attention-deficit/hyperactivity disorder intervention planning. Curr. Psychiatry Rep. 14, 543–551. 10.1007/s11920-012-0297-4 22895892 PMC3724411

[B9] Bidzan-Bluma I. Lipowska M. (2018). Physical activity and cognitive functioning of children: a systematic review. Int. J. Environ. Res. Public Health15:E800. 10.3390/ijerph15040800 29671803 PMC5923842

[B10] Blankenship T. L. O'Neil M. Ross A. Bell M. A. (2015). Working memory and recollection contribute to academic achievement. Learn. Individ. Differ. 43, 164–169. 10.1016/j.lindif.2015.08.020 26644761 PMC4669898

[B11] Bowling A. Slavet J. Miller D. P. Haneuse S. Beardslee W. Davidson K. (2017). Cybercycling effects on classroom behavior in children with behavioral health disorders: an RCT. Pediatrics 139:e20161985. 10.1542/peds.2016-1985 28069663

[B12] Burke R. V. Oats R. G. Ringle J. L. Fichtner L. O. DelGaudio M. B. (2011). Implementation of a classroom management program with urban elementary schools in low-income neighborhoods: does program fidelity affect student behavior and academic outcomes? JESPAR. 16, 201–218. 10.1080/10824669.2011.585944

[B13] Burns R. D. Brusseau T. A. Hannon J. C. (2017). Multivariate associations among health-related fitness, physical activity, and TGMD-3 test items in disadvantaged children from low-income families. Percept. Mot. Skills 124, 86–104. 10.1177/0031512516672118 27703062

[B14] Burns R. D. Fu Y. (2018). Testing the motor competence and health-related variable conceptual model: a path analysis. J. Funct. Morphol. Kinesiol. 3:61 10.3390/jfmk3040061 PMC7739304 33466989

[B15] Burns R. D. Kim Y. Byun W. Brusseau T. A. (2019). Associations of school day sedentary behavior and physical activity with gross motor skills: use of compositional data analysis. J. Phys. Act. Health. 10.1123/jpah.2018-0549. [Epub ahead of print]. 31357260

[B16] Burton A. W. Miller D. E. (1998). Movement Skill Assessment. Champaign, IL: Human Kinetics.

[B17] Cairney J. Velduizen S. Szatmari P. (2010). Motor coordination and emotional-behavioral problems in children. Curr. Opin. Psychiatry 23, 324–329. 10.1097/YCO.0b013e32833aa0aa 20520549

[B18] Cameron C. E. Brock L. L. Murrah W. M. Bell L. H. Worzalla S. L. Grissmer D.. (2012). Fine motor skills and executive function both contribute to kindergarten achievement. Child Dev. 83, 1229–1244. 10.1111/j.1467-8624.2012.01768.x 22537276 PMC3399936

[B19] Castelli D. M. Hillman C. H. Hirsch J. Hirsch A. Drollette E. (2011). FIT Kids: time in target heart zone and cognitive performance. Prev. Med. 52, S55–S59. 10.1016/j.ypmed.2011.01.019 21281671

[B20] Centers for Disease Control and Prevention (2013). Comprehensive School Physical Activity Programs: A Guide for Schools. Atlanta, GA: U.S. Department of Health and Human Services.

[B21] Cohen J. (1988). Statistical Power Analysis for the Behavioral Sciences. Hillsdale, NJ: L. Erlbaum Associates.

[B22] Davis C. L. Cooper S. (2011). Fitness, fatness, cognition, behavior, and academic achievement among overweight children: do cross-sectional associations correspond to exercise trial outcomes? Prev. Med. 52, S65–S69. 10.1016/j.ypmed.2011.01.020 21281668 PMC3164323

[B23] Davis W. E. Burton A. W. (1991). Ecological task analysis: translating movement behavior theory into practice. Adapt. Phys. Activ. Quart. 18, 154–177. 10.1123/apaq.8.2.154

[B24] De Meester A. Stodden D. Brian A. True L. Cardon G. Tallir I.. (2016). Associations among elementary school children's actual motor competence, perceived motor competence, physical activity and BMI: a cross-sectional study. PLoS ONE 11:e0164600. 10.1371/journal.pone.0164600 27736964 PMC5063290

[B25] Donnelly J. E. Hillman C. H. Castelli D. Etnier J. L. Lee S. Tomporowski P. (2016). Physical activity, fitness, cognitive function, and academic achievement in children: a systematic review. Med. Sci. Sports Exerc. 48, 1197–1222. 10.1249/MSS.0000000000000901 27182986 PMC4874515

[B26] Donnelly J. E. Lambourne K. (2011). Classroom-based physical activity, cognition, and academic achievement. Prev. Med. 52, S36–S42. 10.1016/j.ypmed.2011.01.021 21281666

[B27] Finn K. E. Faith M. S. Seo Y. S. (2018). School engagement in relation to body mass index and school achievement in a high-school age sample. J. Obes. 2018:3729318. 10.1155/2018/3729318 30402282 PMC6191957

[B28] Fu Y. Burns R. D. (2018). Gross motor skills and school day physical activity: mediating effect of perceived competence. J. Mot. Learn. Develop. 6, 287–300. 10.1123/jmld.2017-0043

[B29] Gonweld T. Velasques B. Ribeiro P. Machado S. Murillo-Rodriguez E. Ludyga S. (2018). Increasing exercise's effect on mental health: exercise intensity does matter. PNAS. 115, E11890–E11891. 10.1073/pnas.1818161115 30568027 PMC6304946

[B30] Goodway J. D. Robinson L. E. Crowe H. (2010). Gender differences in fundamental motor skill development in disadvantaged preschoolers from two geographical regions. Res. Q. Exerc. Sport 81, 17–24. 10.1080/02701367.2010.10599624 20387395

[B31] Gordon-Larsen P. Nelson M. C. Page P. Popkin B. M. (2006). Inequality in the built environment underlies key health disparities in physical activity and obesity. Pediatrics 117, 417–424. 10.1542/peds.2005-0058 16452361

[B32] Harvey S. P. Lambourne K. Greene J. L. Gibson C. A. Lee J. Donnelly J. E. (2018). The effects of physical activity on learning behaviors in elementary school-aged children. Contemp. Sch. Psychol. 22, 303–312. 10.1007/s40688-017-0143-0 30956895 PMC6428312

[B33] Hillman C. H. Pontifex M. B. Castelli D. M. Khan N. A. Raine L. B. Scudder M. R.. (2014). Effects of the FITKids randomized controlled trial on executive control and brain function. Pediatrics 134, e1063–e1071. 10.1542/peds.2013-3219 25266425 PMC4179093

[B34] Hsieh S. S. Fung D. Tsai H. Chang Y. K. Huang C. J. Hung T. M. (2018). Differences in working memory as a function of physical activity in children. Neuropsychology 32, 797–808. 10.1037/neu0000473 30124313

[B35] Jin Y. Jones-Smith J. C. (2015). Associations between family income and children's physical fitness and obesity in California 2010-2012. Prev. Chronic Dis. 12:E17. 10.5888/pcd12.140392 25674676 PMC4329950

[B36] Lopes L. Santos R. Pereira B. Lopes V. P. (2013). Associations between gross motor coordination and academic achievement in elementary school children. Hum. Mov. Sci. 32, 9–20. 10.1016/j.humov.2012.05.005 23260614

[B37] Loprinzi P. D. Davis R. E. Fu Y.-C. (2015). Early motor skill competence as a mediator of child and adult physical activity. Prev. Med. Rep. 2, 833–838. 10.1016/j.pmedr.2015.09.015 26844157 PMC4721422

[B38] MacDonald M. Lipscomb S. McClelland M. M. Duncan R. Becker D. Anderson K. (2016). Relationships of preschoolers visual-motor and object manipulation skills with executive function and social behavior. Res. Q. Exerc. Sport 87, 396–407. 10.1080/02701367.2016.1229862 27732149 PMC5549668

[B39] Mahar M. T. (2011). Impact of short bouts of physical activity on attention to task in elementary school children. Prev. Med. 52(Suppl 1), S60–S64. 10.1016/j.ypmed.2011.01.026 21281665

[B40] Mahar M. T. Murphy S. K. Rowe D. A. Golden J. Shields A. T. Raedeke T. D. (2006). Effects of a classroom-based program on physical activity and on-task behavior. Med. Sci. Sports Exerc. 38, 2086–2094. 10.1249/01.mss.0000235359.16685.a3 17146314

[B41] Mayorga-Vega D. Aguilar-Soto P. Viciana J. (2015). Criterion-related validity of the 20-M shuttle run test for estimating cardiorespiratory fitness: a meta-analysis. J. Sports Sci. Med. 14, 536–547. 26336340 PMC4541117

[B42] Michem K. J. Young K. R. West R. P. Benyo J. (2001). CWPASM: a class-wide peer assisted self-management program for general education classrooms. Educ. Treat. Children 24, 111–140.

[B43] Moller A. C. Forbes-Jones E. Hightower A. D. (2008). Classroom age composition and developmental change in 70 urban preschool classrooms. J. Educ. Psychol. 100, 741–753. 10.1037/a0013099

[B44] Morley D. Till K. Ogilvie P. Turner G. (2015). Influences of gender and socioeconomic status on the motor proficiency of children in the UK. Hum. Mov. Sci. 44, 150–156. 10.1016/j.humov.2015.08.022 26342797

[B45] Nuttall F. Q. (2011). Body mass index: obesity, BMI, and health: a critical review. Nutr. Today 50, 117–128. 10.1097/NT.0000000000000092 27340299 PMC4890841

[B46] Pellegrini A. D. Davis P. D. (1993). Relations between children's playground and classroom behavior. Brit. J. Educ. Psychol. 63, 88–95. 10.1111/j.2044-8279.1993.tb01043.x 8466835

[B47] Playford C. J. Dibben C. Williamson L. (2017). Socioeconomic disadvantage, fetal environment and child development: linked Scottish administrative records based study. Int. J. Equity Health 16:203. 10.1186/s12939-017-0698-4 29166913 PMC5700527

[B48] Puterman E. O'Donovan A. Adler N. E. Tomiyama A. J. Kemeny M. Wolkowitz O. M.. (2011). Physical activity moderates stressor-induced rumination on cortisol reactivity. Psychosom. Med. 73, 604–611. 10.1097/PSY.0b013e318229e1e0 21873586 PMC3167008

[B49] Rathvon N. (2008). Effective School Interventions: Evidence-Based Strategies for Improving Student Outcomes (2nd ed). New York, NY: Guilford Press.

[B50] Reardon S. F. (2011). “The Widening Academic Achievement Gap Between the Rich and the Poor: New Evidence and Possible Explanations,” in Whither Opportunity? Rising Inequality and the Uncertain Life Chances of Low-Income Children, eds Murnane R. Duncan G. (New York, NY: Russell Sage Foundation), 1–49.

[B51] Robinson L. E. Palmer K. K. Bub K. L. (2016). Effect of the children's health activity motor program on motor skills and self-regulation in head start preschoolers: an efficacy trial. Front. Pub. Health 4:173. 10.3389/fpubh.2016.00173 27660751 PMC5014876

[B52] Robinson L. E. Stodden D. F. Barnett L. M. Lopes V. P. Logan S. W. Rodrigues L. P.. (2015). Motor competence and its effect on positive developmental trajectories of health. Sports Med. 45, 1273–1284. 10.1007/s40279-015-0351-6 26201678

[B53] Sanabria D. Luque-Casado A. Perales J. C. Ballester R. Ciria L. F. Huertas F. (2019). The relationship between vigilance capacity and physical and exercise: a mixed-effects multi-study analysis. PeerJ. 7:e7118 10.7717/peerj.7118 31205826 PMC6556370

[B54] Scott T. M. Park K. L. Swain-Bradway J. Landers E. (2007). Positive behavior support in the classroom: facilitating behaviorally inclusive learning environments. Int. J. Behav. Consult. Therap. 3, 223–235. 10.1037/h0100800

[B55] Silva-Santos S. Santos A. Duncan M. Vale S. Mota J. (2019). Association between moderate and vigorous physical activity and gross motor coordination in preschool children. J. Mot. Learn Develop. 7, 273–285. 10.1123/jmld.2017-0056

[B56] Stodden D. F. Gao Z. Goodway J. D. Langendorfer S. J. (2014). Dynamic relationships between motor skill competence and health-related fitness in youth. Pediatr. Exerc. Sci. 26, 231–241. 10.1123/pes.2013-0027 25111159

[B57] Stodden D. F. Goodway J. D. Langendorfer S. J. Roberton M. A. Rudisill M. E. Garcia C. (2008). A developmental perspective on the role of motor skill competence in physical activity: an emergent relationship. Quest 60, 290–306. 10.1080/00336297.2008.10483582

[B58] Stylianou M. Kulinna P. H. van der Mars H. Mahar M. T. Adams M. A. Amazeen E. (2016). Before-school running/walking club: effects on student on-task behavior. Prev. Med. Rep. 3, 196–202. 10.1016/j.pmedr.2016.01.010 27419015 PMC4929141

[B59] Tarp J. Child A. White T. Westgate K. Bugge A. Grontved A.. (2018). Physical activity intensity, bout-duration, and cardiometabolic risk markers in children and adolescents. Int. J. Obes. 42, 1639–1650. 10.1038/s41366-018-0152-8 30006582 PMC6160399

[B60] Taylor R. W. Gold E. Manning P. Goulding A. (1997). Gender differences in body fat content are present well before puberty. Int. J. Obes. Relat. Metab. Disord. 21, 1082–1084. 10.1038/sj.ijo.0800522 9368835

[B61] Ulrich D. A. (2000). Test of Gross Motor Development. (2nd ed.). Austin, TX: PRO-ED.

[B62] van der Mars H. (1989). “Basic recording tactics,” in Analyzing Physical Education and Sport Instruction, eds Darst P. W. Zakrajsek D. B. Mancini V. H. (Champaign, IL: Human Kinetics), 53–80.

[B63] Winter B. Breitenstein C. Mooren F. C. Voelker K. Fobker M. Lechtermann A.. (2007). High impact running improves learning. Neurobiol. Learn. Mem. 87, 597–609. 10.1016/j.nlm.2006.11.003 17185007

[B64] Zeng N. Ayyub M. Sun H. Wen X. Xiang P. Gao Z. (2017). Effects of physical activity on motor skills and cognitive development in early childhood: a systematic review. Biomed Res. Int. 2017:2760716. 10.1155/2017/2760716 29387718 PMC5745693

